# Methylomics of nitroxidative stress on precancerous cells reveals DNA methylation alteration at the transition from *in situ* to invasive cervical cancer

**DOI:** 10.18632/oncotarget.18370

**Published:** 2017-06-06

**Authors:** Po-Hsuan Su, Yao-Wen Hsu, Rui-Lan Huang, Yu-Chun Weng, Hui-Chen Wang, Yu-Chih Chen, Yueh-Ju Tsai, Chiou-Chung Yuan, Hung-Cheng Lai

**Affiliations:** ^1^ Translational Epigenetics Center, Shuang Ho Hospital, Taipei Medical University, Taipei, Taiwan; ^2^ Department of Obstetrics and Gynecology, Shuang Ho Hospital, Taipei Medical University, Taipei, Taiwan; ^3^ Graduate Institute of Life Sciences, National Defense Medical Center, Taipei, Taiwan; ^4^ Department of Obstetrics and Gynecology, School of Medicine, College of Medicine, Taipei Medical University, Taipei, Taiwan; ^5^ Division of Research and Analysis, Food and Drug Administration, Ministry of Health and Welfare, Taipei, Taiwan; ^6^ Department of Clinical Pharmacology, Xiangya Hospital, Central South University, Changsha, P. R. China; ^7^ Institute of Clinical Pharmacology, Central South University, Hunan Key Laboratory of Pharmacogenetics, Changsha, P. R. China

**Keywords:** chronic inflammation, nitric oxide, nitroxidative stress, gene specific hypermethylation

## Abstract

Epigenetic dysregulation is important in cervical cancer development, but the underlying mechanism is largely unknown. Increasing evidence indicates that DNA methylation is sensitive to changes in microenvironmental factors, such as nitric oxide (NO) in the chronic inflammatory cervix. However, the epigenomic effects of NO in cancer have not been investigated. In this study, we explored the methylomic effects of nitroxidative stress in HPV-immortalized precancerous cells. Chronic NO exposure promoted the acquisition of malignant phenotypes such as cell growth, migration, invasion, and anchorage-independent growth. Epigenetic analysis confirmed hypermethylation of *PTPRR*. Whole-genome methylation analysis showed *BOLA2B*, *FGF8*, *HSPA6*, *LYPD2*, and *SHE* were hypermethylated in cells. The hypermethylation *BOLA2B*, *FGF8*, *HSPA6*, and *SHE* was confirmed in cervical scrapings from invasive cancer, but not in CIN3/CIS, CIN2 and CIN1 (*p*=0.019, 0.023, 0.023 and 0.027 respectively), suggesting the role in the transition from *in situ* to invasive process. Our results reveal that nitroxidative stress causes epigenetic changes in HPV-infected cells. Investigation of these methylation changes in persistent HPV infection may help identify new biomarkers of DNA methylation for cervical cancer screening, especially for precancerous lesions.

## INTRODUCTION

Epigenetic alteration is a critical driving force in cervical cancer development [1]. Our previous works and others have discovered many genes DNA-methylated in cervical cancer [2–5]. The application of DNA methylation as a biomarker for cervical cancer screening, triage of high risk human papilloma virus (hrHPV) infection or mildly abnormal cytology is under intensive investigations [3–5]. HPV infection is the cause of cervical cancer and has been reported to induce epigenetic changes [6]. HPV oncoprotein E7 can interact with DNA methyltransferase 1 (DNMT1) which is a major enzyme that maintains DNA methylation patterns during replication. HPV oncoproteins E6 can increase the expression of DNMT1 [7]. However, the mechanism leading to these DNA methylation changes during cervical carcinogenesis remains largely unknown.

Chronic inflammation is also a culprit of cancer development [8]. Accumulating evidence also suggest chronic inflammation may cause epigenetic changes in cancers, such as *Helicobacter pylori* infection and gastric carcinoma [9] and hepatitis B or C virus infection and hepatocellular cancer [10]. In cervical cancer, the cervical tissue inflammation was associated with high grade lesions in oncogenic HPV-infected women [11]. The expression of IL-8, IL-10 and nitric oxide (NO) in cervical intraepithelial neoplasia (CIN) lesions is higher than in normal cervix [12]. However, the epigenetic effects of inflammatory factors in HPV-initiated cervical cancer remain largely unknown.

Oxidative stress in chronic inflammation contributes to cervical cancer development [13]. Epidemiological data show that virus infection, smoking, multiparity, long-term use of oral contraceptives, and sexually transmitted infections are risk factors for cervical cancer [14]. All of these factors also increase NO in the cervical microenvironment [13, 15]. NO induced nitrative DNA damage is important in inflammation related carcinogenesis [16]. The level of NO is higher in women with a hrHPV compared with those with a low risk HPV infection [17] Tumor-associated macrophages in cervical cancer also produce NO in the tumor microenvironment [18]. All these evidence suggest that NO in an important modulator in cervical carcinogenesis. The epigenetic effects of NO in cervical cancer have never been investigated.

In this study, we explored the DNA methylation effects of NO exposure in HPV-immortalized precancerous cells. We hypothesized that chronic inflammation with NO exposure can cause DNA methylation alterations and promote the acquisition of malignant phenotypes. The inflammation-mediated DNA methylation may be used as biomarkers for clinical applications.

## RESULTS

### Chronic NO exposure increased the expression of the malignant phenotypes in cervical precancerous cell lines

**Figure 1:**
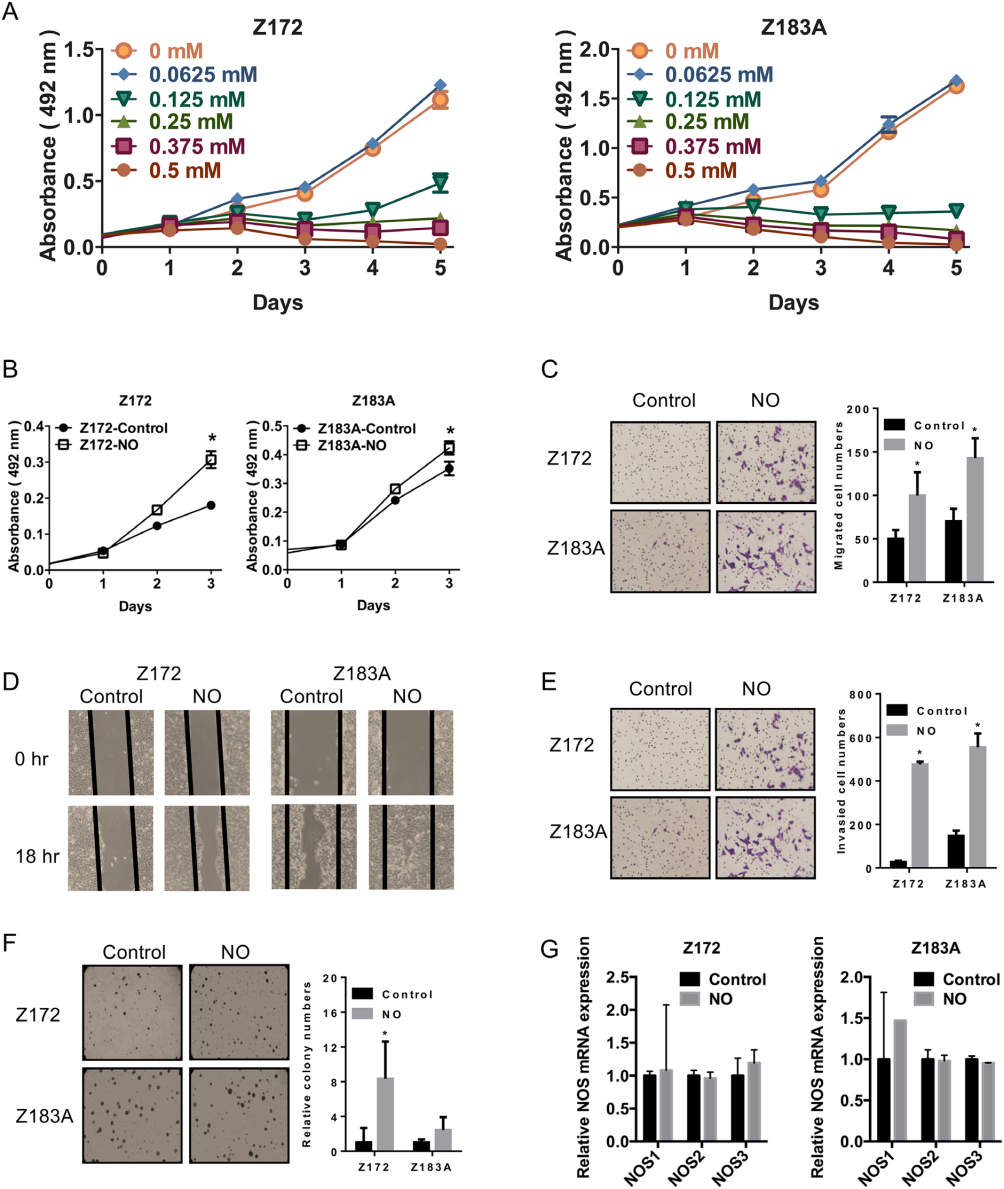
Effects of NO on malignant phenotypes in Z172 and Z183A cell lines **(A)** NO inhibited cell proliferation in a dose-dependent manner in Z172 and Z183A cell lines (DETA-NO concentration: 0.0625 mM–0.5 mM). Chronic NO exposure increased cell growth, colony formation, migration, and invasion ability in HPV-immortalized cell lines. **(B)** Cell growth was assayed by the MTS assay. Migration assay assayed in a transwell system without coating **(C)** and wound-healing assay **(D)**. Cell invasion was measured in the transwell system coated with Matrigel **(E)**. **(F)** Colony formation in soft agar is shown on the left. **(G)** qPCR analysis of NOS mRNA in Z172 and Z183A after NO treatment. Quantification relative to controls is shown on the right. *p<0.05.

To examine the potential biological effects of NO in cervical cancer progression, we generated a model of chronic low-dose NO exposure in precancerous cells. We titrated the NO donor (DETA-NO) concentration and measured the proliferation of the cervical precancerous cell lines, Z172 and Z183A. Z172 and Z183A are full length-HPV immortalized cervical epithelial cells which were immortalized by HPV16 and HPV18 respectively [19]. The regulation mechanism of HPV viral genes, such as E2 protein and long control region, remain in the cells. These cells are more similar to the real physical situation of HPV infected cervical epithelial cells in precancerous stage. The previous study showed the injection of Z172 and Z183A did not form tumor *in vivo* [20]. This is different from the cell lines used in most studies that transformed by SV40 promoter-driven HPV E6/E7. NO treatment resulted in a dose-dependent inhibition of proliferation (Figure 1A). We used 0.0625 mM DETA-NO for further experiments because this concentration did not show significant effects on cell growth (Figure 1A).

After treatment with 0.0625 mM NO for four weeks, the phenotypes of these immortalized precancerous cell lines were examined. Both cell lines acquired significant signs of malignant phenotypes such as increased cell proliferation, migration, invasion, and anchorage-independent growth *in vitro* (Figure 1B–1F). NO is synthesized from L-arginine by a family of nitric oxide synthase (NOS) enzymes including NOS1 (neuronal NOS), NOS2 (inducible NOS) and NOS3 (endothelial NOS) [21]. The human cervical cancer tissue expressed NOS2 [22]. NO-mediated mutagenesis and increased levels of NO have been observed in cervical cancer, but the association of NO and NOS in cervical cancer malignancy was not studied. Hence, we analyze the expression NOS in cells that gained malignant phenotypes after NO treatment. The result showed the expression of NOS did not change significantly after NO treatment (Figure 1G).

### NO induced gene-specific hypermethylation in Z172 and Z183A cervical precancerous cell lines

**Figure 2:**
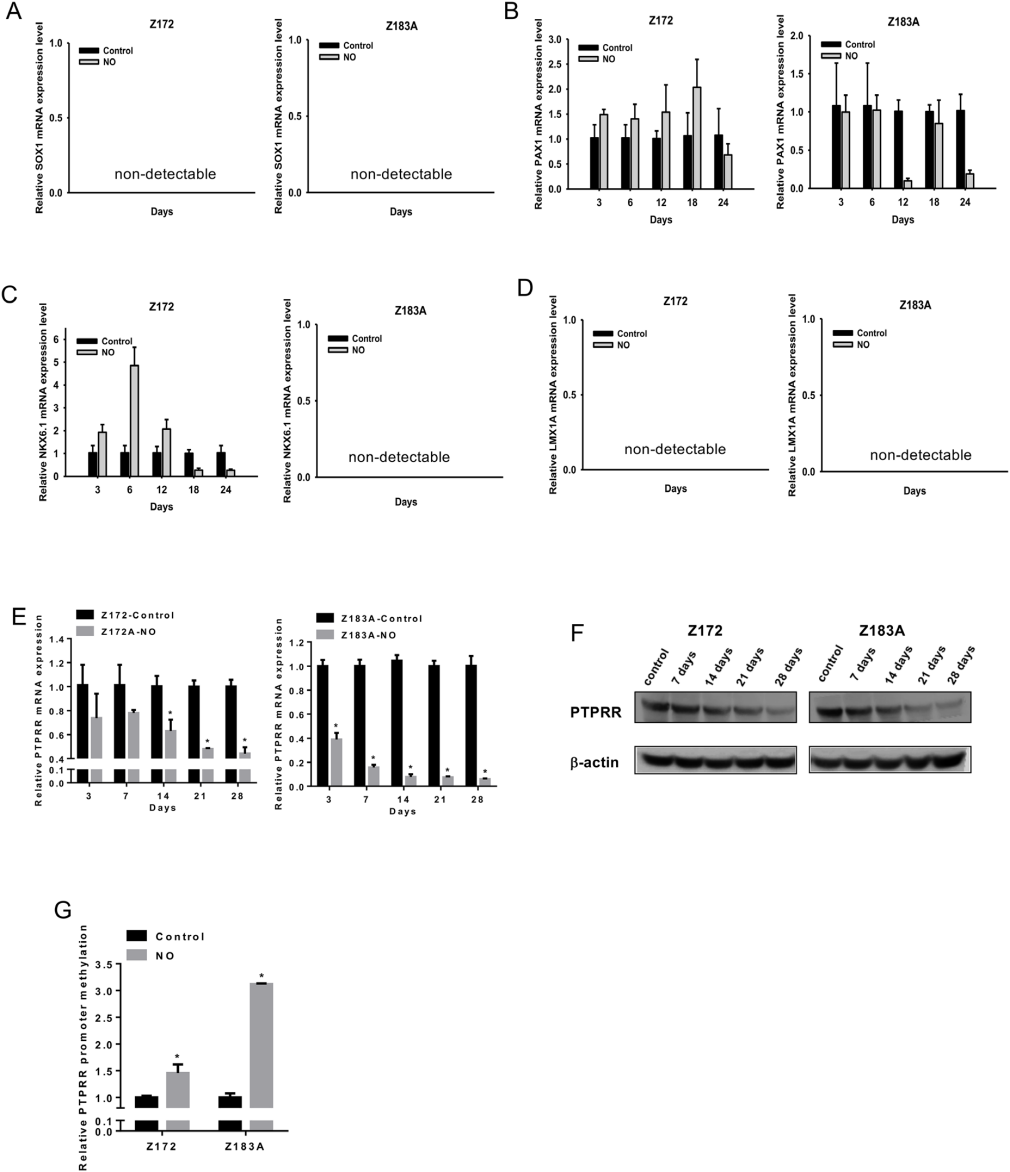
Inhibition of PTPRR gene expression and increase in promoter DNA methylation after NO treatment in HPV-immortalized cell lines mRNA Expression of SOX1 **(A)**, PAX1 **(B)**, NKX6.1 **(C)**, LMX1A **(D)**, and PTPRR **(E)** after NO treatment in HPV-immortalized cell lines. qPCR analysis of mRNA **(E)** and western blot analysis of protein **(F)** showed reduced mRNA and protein of PTPRR after NO treatment in Z172 and Z183A cell lines. qMS-PCR showed increased methylation of the PTPRR promoter after NO treatment **(G)**. *p<0.05.

After determining the acquisition of malignant phenotypes after chronic low-dose NO exposure in these cell lines, we asked whether such changes were accompanied by DNA methylation changes. To test whether NO exposure could induce gene-specific hypermethylation in HPV infected cells, we measured the expression of five methylation-silenced genes (SOX1, PAX1, NKX6.1, LMX1A and PTPRR). These genes were methylation-silenced in invasive cervical cancer that previously discovered by methylomic approaches [2, 23, 24]. Because the expression of these genes in precancerous cell lines was unknown, therefore, we analysis the expression of theses gene in Z172 and Z183A after NO treatment. The expression of PTPRR not others changes after NO stress (Figure 2A-2E). We confirmed the downregulation of PTPRR mRNA and protein expression, and increased PTPRR promoter methylation in both cell lines (Figure 2E-2G). PTPRR is a tyrosine phosphatase that can inhibit the phosphorylation status of ERK 1/2, the expression of the transcription factor AP1 and HPV oncogenes E6/E7. PTPRR inhibits tumor formation and metastasis *in vivo*. Promoter methylation of PTPRR was found in invasive cervical cancer cell lines and tissues [2]. In Z172 cell, the expression of PAX1 and NKX6.1 were biphasic. The similar phenomenon was found in immune cells. NO has a biphasic effect on NF-κB activity in murine macrophages and hence the target of NF-κB, such as NOS2 and COX-2, was biphasic expression [6]. In the 3k bps upstream from the transcription start site of PAX1 and NKX-6.1, there were three and five NF-κB-binding sites respectively. Therefore, the biphasic expression of PAX1 and NKX6.1 may cause by NF-κB. This possibility deserves further investigations.

### Methylomic analysis of Z172 and Z183A cells after chronic NO exposure

**Figure 3:**
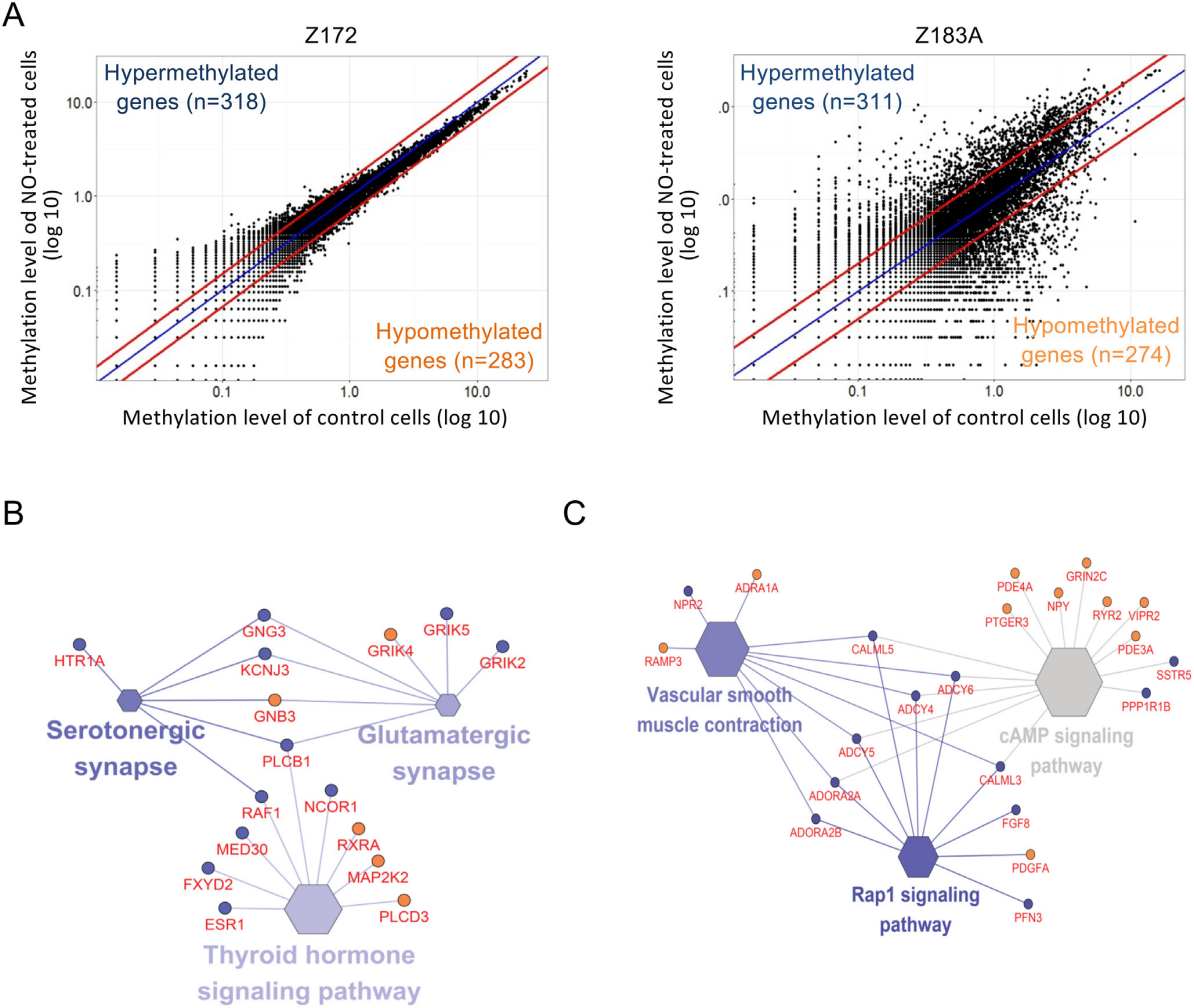
NO influences on DNA methylation changes in precancerous cells **(A)** displayed the differential methylation analysis between NO treated and control in Z172 and Z183A cells. One dot present a CpG site. The outsider dots of red lines showed the 1.5-fold changes of methylation level in Z172 and 2-fold changes of methylation level in Z183A. The enriched signaling pathways presented genes of methylation changes in NO-treated Z172 cell **(B)** and Z183A cell **(C)**. The size and color grading of hexagon represent the enrichment significance of each term. The larger size of hexagon showed the higher significance, the darker color showed higher percentage of genes in a singling pathway. Blue circle: hypermethylated gene; red circle: hypomethylated gene.

To identify the genes modulated by NO in HPV-infected cells, we used a methylomics approach using MethylCap-seq (Figure 3A). Z183, which is an HPV18 infected cell line, demonstrated a more diverse pattern of methylome than the HPV16-infected Z172. Using a 1.5-fold threshold, there were 345 genes hypermethylated and 366 genes hypomethylated in Z172; and using a 2-fold threshold, there were 357 genes hypermethylated and 317 genes hypomethylated in Z183A. Using network analysis, we found changes of methylations in genes involved in thyroid hormone signaling pathway, serotonergic synapse and glutamatergic synapse in Z172 (Figure 3B) and cAMP signaling pathway, Rap1 signaling pathway and vascular smooth muscle contraction in Z183A (Figure 3C).

### Validation of candidate genes in cell lines and clinical specimens

**Figure 4:**
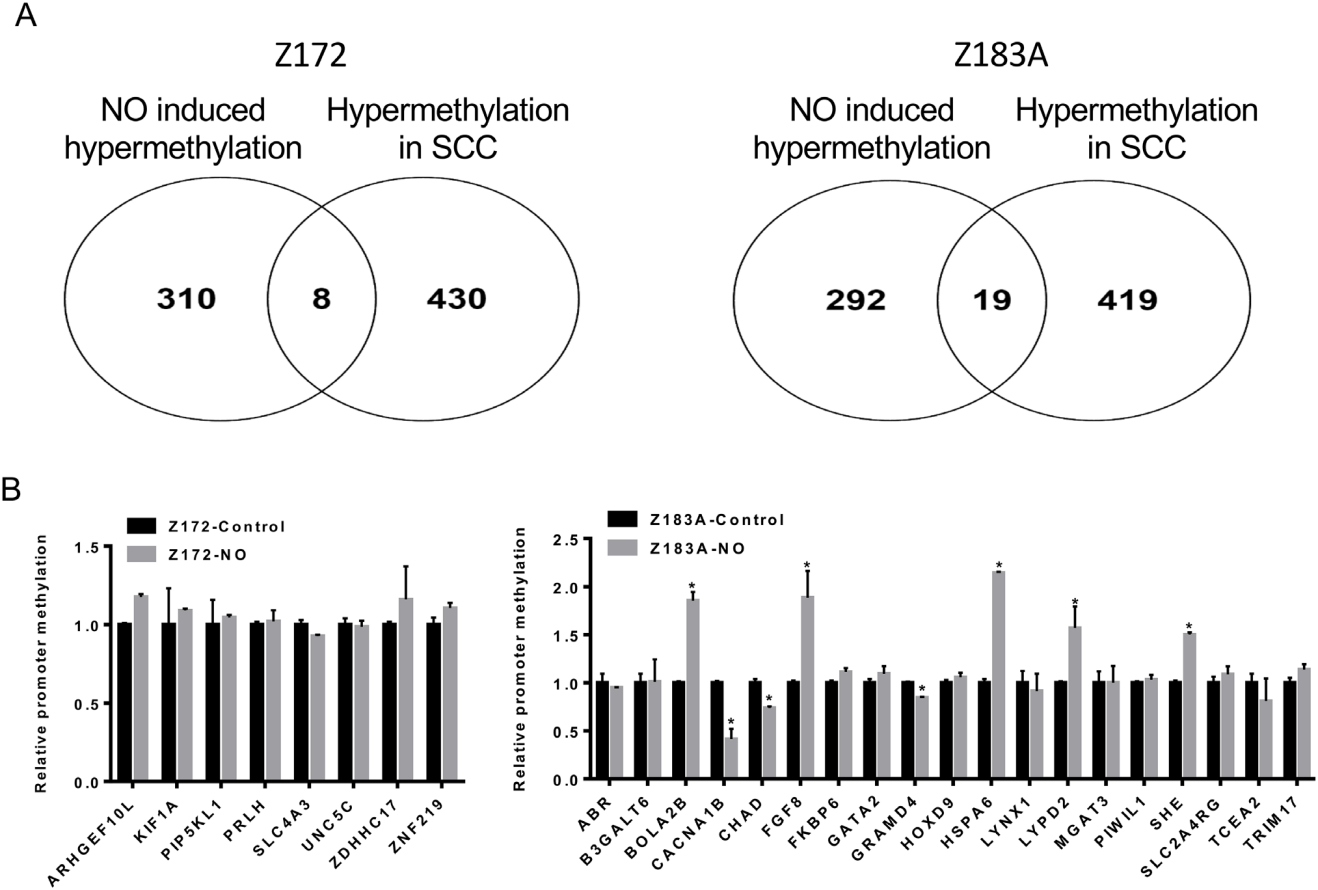
The validation of the methylation changes in cell lines The assembly of methylomics results from cell lines and human cancer tissues. 8 and 19 genes hypermethylated in Z172 and Z183, respectively after NO treatment **(A)**. The validation of 27 genes in Z172 and Z183A **(B)**. *p<0.05.

**Figure 5:**
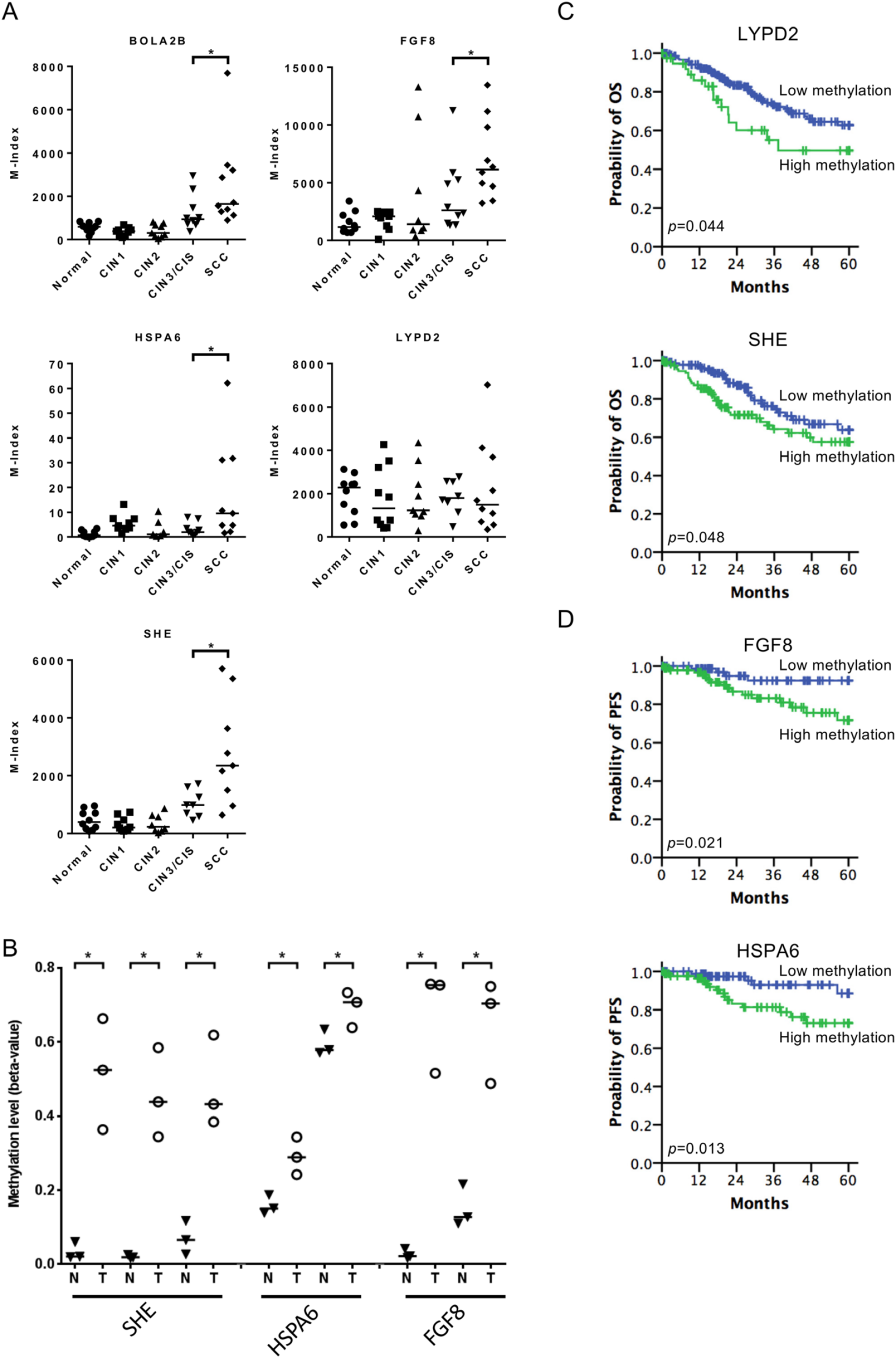
Application of nitrative-induced methylation genes in clinical specimens **(A)** Hypermethylation genes (*BOLA2B*, *FGF8*, *HSPA6*, and *SHE*) were tested in DNA from cervical scrapings of human cervical lesions (*p*=0.019, 0.023, 0.023 and 0.027 respectively). The median for each group is indicated by a horizontal bar. **(B)** Methylation status of SHE (probe: cg18081940, cg02457680 and cg05637536), HSPA6 (probe: cg23691642 and cg03524848) and FGF8 (probe: cg25209842 and cg06169131) in paired normal tissue and cancer tissue in TCGA database. Kaplan–Meier plots of the probability of OS and PFS in TCGA cervical cancer patients. **(C)** OS were stratified by the methylation status of LYPD2 (probe: cg16733117) and SHE (probe: cg24889998). **(D)** PFS were stratified by the methylation status of FGF8 (probe: cg25209842) and HSPA6 (probe: cg00769882). *p<0.05.

To validate the methylation changes of cell lines in human tissues, we merged methylomics results from cell lines and human cancer tissues we previously published [25]. We found 8 and 19 genes hypermethylated in Z172 and Z183, respectively after NO treatment (Figure 4A). These 27 genes were further verified by quantitative methylation-specific PCR (qMS-PCR). Five genes including BolA family member 2B (BOLA2B), fibroblast growth factor 8 (FGF8), heat shock 70kDa Protein 6 (HSPA6), LY6/PLAUR Domain Containing 2 (LYPD2) and Src Homology 2 Domain Containing E (SHE) were hypermethylated in cell lines after NO treatment (Figure 4B). These hypermethylated genes were tested in DNA from cervical scrapings of human cervical lesions. The methylation levels of BOLA2B, FGF8, HSPA6, LYPD2 and SHE were low in normal cervix, low grade lesions and CIN3/CIS, but significantly higher in invasive cancer (Figure 5A). We analyzed the methylation status of these genes in TCGA database and found the methylation of SHE, HSPA6 and FGF8 were lower in normal tissue then cancer tissue (Figure 5B). Moreover, hypermethylation of LYPD2 and SHE were associated with poor overall survival (OS); hypermethylation of FGF8 and HSPA6 were associated with poor progression free survival (PFS) in patients with cervical cancer (Figure 5C and 5D).

## DISCUSSION

The present study for the first time demonstrates that chronic nitroxidative inflammation can cause epigenomic effects in HPV infected cells. NO exposure causes hypermethylation at some specific gene promoters and global hypomethylation, especially of pericentromeric repetitive sequences. NO is synthesized by nitric oxide synthase (NOS) enzymes, it serves as a signaling molecule in various physiological processes. Dysregulated NO synthesis has been implicated as causal or contributing to pathophysiological conditions including cancer. Infection with hrHPV induces expression of nitric oxide synthase (NOS) in the human uterine cervix and increased levels of NO in their cervical fluid [26]. Taken together, chronic nitroxidative stress-mediated epigenomic changes is a driving force for hrHPV-infected precancerous cells to invasive cancer.

BOLA2B is involved in iron homeostasis, can be increased by Myc in liver cell dysplasia [27]. FGF8 is a member of the fibroblast growth factor (FGF) family is involved in tumor growth and invasion in nasopharyngeal carcinoma [28]. 35 human Ly6/uPAR family members have been identified. They regulate progression of inflammation, complement activity, neuronal activity, angiogenesis, wound healing, and cancer growth [29]. But the function of LYPD2 is still unknown. Proteins contain Src homology 2 domain are important in many signal transduction pathways [30], however, the function of SHE was never studied. HSPA6 in upregulated in hepatocellular carcinoma and breast and is associated with poor outcomes in hepatocellular carcinoma [31, 32]. According to the result of NGS, BOLA2B was methylated at 600-800 bps upstream from the transcription start site (TSS); FGF8 was methylated at 600-800 bp upstream of the TSS; HSPA6 was methylated at 400-800 bp upstream of the TSS; LYPD2 was methylated at 0-200 bp upstream of the TSS; SHE was methylated at 800-1000 bp upstream of the TSS. Putative binding motif analysis (Promo) in these regions indicated the presence of numerous putative glucocorticoid receptor alpha (GR-alpha) binding motifs in these genes. GR-alpha has transcriptional repression activity and can be upregulated by nitric oxide [33]. These five genes are not studied in cervical cancer.

From the clinical point of view, current DNA methylation biomarkers for cervical cancer screening are promising but still with limitations. Generally, the sensitivities of DNA methylation biomarkers for the purpose of screening are moderate [34]. The sensitivity for invasive cancer is high, however, the detection of CIN lesions is still not satisfactory [35]. The discovery of genes methylated in precancerous lesions to improved molecular cervical cancer screening in imperative. The present study provides the evidence that chronic inflammation such as nitroxidative stress can cause epigenetic changes in HPV-infected cells. Limited by the cell models, which is a CIN3 equivalent cells, BOLA2B, FGF8, HSPA6, LYPD2 and SHE hypermethylation discovered in the present study can be detected in invasive not precancerous cervical scrapings. Further methylomic investigations of oxidative stress in earlier HPV infected cells or persistent HPV infection patients may lead to the discovery of new genes for precancerous screening.

In summary, our results demonstrated the epigenomic effects of chronic nitroxidative stress in HPV-immortalized precancerous cell lines. Further elucidation of inflammation induced epigenomics in persistent HPV infection may help identify potential DNA methylation biomarkers for early detection of cervical cancer.

## MATERIALS AND METHODS

### Clinical samples

A hospital-based, retrospective, case-control study was conducted on 50 patients, including women who had a normal uterine cervix (n = 10), CIN1 (n = 10), CIN2 (n = 10), CIN3/carcinoma *in situ* (CIS) (n = 10) and squamous cell carcinoma (SCC) (n = 10) of the uterine cervix diagnosed according to histologic reports. Cervical scrapings were used for the analysis. A cervical brush (Pap Brush; Young Ou Company, Ltd., Yongin City, Korea) was used to collect cervical scrapings. The brush was preserved in phosphate-buffered saline solution at 4°C until DNA extraction. All patients were diagnosed, treated, and had their tissues banked at the National Defense Medical Center, Taipei, Taiwan. The final diagnosis was made by tissue-proven pathology rather than cytology except for controls. Controls were recruited from healthy women who underwent routine Pap screening. Informed consent was obtained from all patients and controls. Exclusion criteria included pregnancy, chronic or acute systemic viral infections, a history of cervical neoplasia, skin or genital warts, an immunocompromised state, the presence of other cancers, or a history of surgery to the uterine cervix. The Institutional Review Board of the Tri-Service General Hospital approved this study. We used the DNA methylation data obtained from cervical cancer patients from the The Cancer Genome Atlas (TCGA) data portal. The methylation dataset of the Illumina HumanMethylation450 BeadChip was used to perform a survival analysis based on the information obtained from the TCGA data portal.

### Cell lines, culture conditions, and NO treatment

The human cervical precancerous cell lines Z172 and Z183A were cultured in Dulbecco’s modified Eagle’s medium (DMEM) with 10% (w/v) Nu-Serum (BD Bioscience, San Jose, CA, USA), penicillin at 100 U/ml (Thermo Fisher Scientific), streptomycin at 100 μg/ml (Thermo Fisher Scientific), and hydrocortisone at 50 ng/ml (Sigma-Aldrich) [20]. For NO treatment, cells were incubated in fresh medium containing the NO donor (Z)-1-[N-(2-aminoethyl)-*N*-(2-ammonioethyl)amino]diazen-1-ium-1,2-diolate (DETA-NO). The cell culture medium was changed every day. The concentration of NO in supernatants was measured by the Griess method (Promega), which measures the stable NO metabolites NO_2_^–^ and NO_3_^–^ [36].

### RNA extraction, cDNA synthesis, and quantitative real-time PCR (qPCR)

Total RNA was isolated from each sample using a Qiagen RNeasy kit (Qiagen). An additional DNase I digestion procedure (Qiagen) was included in the isolation of RNA to remove contaminating DNA according to the manufacturer’s protocol. RNA was reverse transcribed to cDNA using the Super Script III first-strand synthesis system for RT-PCR (Thermo Fisher Scientific) according to the manufacturer’s protocol. Oligo(dT) was used as the primer for RNA. qPCR was performed using LightCycler 480 SYBR Green I Master (Roche) in a LightCycler 480 Real-Time PCR System (Roche). Relative gene expression was determined based on the threshold cycles (Ct) of the genes of interest and the internal reference gene, GAPDH. The average Ct value of the GAPDH gene was subtracted from the average Ct value of the gene of interest for each sample, and the fold change was calculated (two-fold per ΔCt). All values are expressed as mean ± SEM. The primers used in this study are shown in Supplementary Table 1.

### DNA extraction, bisulfite modification, quantitative methylation-specific PCR (qMS-PCR)

Genomic DNA was extracted from the cultured cells, scraped cells, or tumor tissues with the QIAmp DNA Mini Kit (QIAGEN). Bisulfite modification was performed using a CpGenome Fast DNA Modification Kit (Millipore, Bedford, MA, USA). Modified DNA was stored at -80°C. qMS–PCR was performed in an SYBR Green system using the LightCycler 480 Real-Time PCR System (Roche). The type II collagen gene (*COL2A*) was used as the internal reference gene. The DNA methylation level was assessed as the methylation index (M-index), 10 000 × 2([Cp of *COL2A*] – [Cp of gene] [37]. Samples with a Cp value for *COL2A* greater than 36 were be defined as detection failures and were discarded. Primers for qMS-PCR and bisulfite sequencing will be provided upon request.

### Cell proliferation assay

Cells were seeded in 96-well plates at a density of 4000 cells/well. On days 0, 1, 2, 3, and 4, cell viability was measured using an MTS assay (C CellTiter 96 AQueous Non-Radioactive Cell Proliferation Assay (Promega) according to the manufacturer’s instructions. Briefly, the MTS reagent (20 μl/ well) was added to 100μl of medium containing cells in each well of a 96-well plate, and the plate was incubated for 2 hr. For the colorimetric analysis, the absorbance at 490 nm was recorded using a microplate reader. Experiments were repeated four times.

### Cell migration assay and invasion assays

Cell migration was assessed using the scratch wound-healing assay. Cells were plated into a 6 cm culture dish, grown to confluence, and then scratched with a sterile 10 μl pipette tip. The suspended cells were removed by washing, and the dish was photographed immediately after scratching (day 0) and again after 24 h. Cell migration was quantified by measuring the area into which cells had migrated, using ImageJ software.

Cell invasion was measured in the Transwell system (BD Bioscience). The chamber membrane was coated with Matrigel (BD Bioscience). Twenty thousand cells were suspended in culture medium without serum and seeded in the upper chamber, and the same medium with serum was added to the lower chamber. After incubation 24 h, cells that had permeated the Matrigel and migrated to the lower surface of the filter were fixed with methanol and stained with Giemsa’s azur eosin methylene blue solution (Merck). The chambers were photographed under a microscope, and the cells in each chamber were counted. The number of transfected cells that migrated through the Matrigel was normalized by the mean cell counts of the control cell lines.

### Immunoblot analysis

Cells were washed twice with PBS and lysed with M-PER (Thermo) supplemented with protease inhibitors. Soluble proteins were boiled in SDS-sample buffer, separated by SDS-PAGE, and transferred to PVDF membranes (Millipore). The membranes were incubated with different primary antibodies and suitable secondary antibodies. Antigen–antibody complexes were detected using Immobilon Western Chemiluminescent HRP Substrate or Immobilon Western AP Substrate (both from Millipore). The antibodies used in the immunoblot analysis were anti-PTPRR (ab88598, Abcam) and anti-β-actin (ab8226, Abcam).

### Differential methylation analysis

Methylomics analysis was performed using methyl-DNA binding domain capture coupled with next-generation sequencing (MethylCap-seq). We calculated the DNA methylation level using the uniquely mapped reads and normalized these to the total read number of each sample [38]. We focused the methylation changes at a 2000 bp region spanning 1000 bp upstream and downstream of the transcription start site (TSS) of the coding genes (reference genome: hg19 from UCSC). We excluded the TSS regions in the sex chromosome, and the remaining 26,751 TSS regions were analyzed in this study. We selected 1.5-fold changes in the methylation level in Z172 cells and 2-fold changes in the methylation level in Z183A cells.

### Network groupings based on functional annotation of the enrichment analysis

To evaluate network groupings based on the functional annotation, we used the ClueGO tool, a Cytoscape plugin [39]. ClueGO facilitates the visualization of functionally related genes by displaying them as a clustered network. The enrichment analysis was based on the two-sided hypergeometric test with a Bonferroni correction and 0.5 kappa score in KEGG (version 10.02.2016).

### Statistical analysis

The Mann–Whitney *U* test was used to compare cell proliferation, migration, invasion, anchorage-independent growth (AIG), and relative RNA expression and promoter methylation. Kaplan–Meier analysis and log-rank tests were used to estimate the survival distributions and to compare differences between the curves for progression-free survival (PFS) and overall survival (OS). All analyses were two-sided, and *p*-values <0.05 were regarded as significant. All statistical calculations were performed using the statistical package SPSS version 20.0 for Windows (IBM Corp.).

## SUPPLEMENTARY MATERIALS TABLE


