# Supplementary material for: Methylomics of nitroxidative stress on precancerous cells reveals DNA methylation alteration at the transition from in situ to invasive cervical cancer
Source: Oncotarget. 2017 Jun 6;8(39):65281–91. doi: 10.18632/oncotarget.18370 (PMC5630330; doi:10.18632/oncotarget.18370)
Supplement: Supplementary file 1 [file oncotarget-08-65281-s001.pdf]

Methylomics of nitroxidative stress on precancerous cells reveals DNA methylation alteration at the transition from *in situ* to invasive cervical cancer

SUPPLEMENTARY INFORMATION

Supplementary Table 1: Primers used in the present study

Gene	Forward primer	Reverse primer
GAPDH	ACCCACTCCTCCACCTTTGACG	TCTCTTCCTCTTGTGCTCTTG
SOX1	AGACCTAGATGCCAACAATTGG	GCACCACTACGACTTAGTCCG
LMX1A	GCTGCTTCTGCTGCTGTGTCT	ACGTTTGGGGCGCTTATGGTC
PAX1	CCTACGCTGCCCTACAACCACATC	TCACGCCGGCCCAGTCTTCCATCT
NKX6.1	CACACGAGACCCACTTTTTCC	CCCAACGAATAGGCCAAACG
PTPRR	CATGCTGGATGTAGAAGAAGACA	AACACCCTGTTCTACCTATTCCTG
NOS1	ACAAGGTCCGATTCAACAGC	AGGCCAAAAACTGAGAACCTC
NOS2	TCCCGAAGTTCTCAAGGCAC	TTCTTCACTGTGGGGCTTGC
NOS3	CCCCAGCATCCCTACTCC	ACCTCCCAGTTCTTCACACG
